# Multispecies probiotics complex improves bile acids and gut microbiota metabolism status in an *in vitro* fermentation model

**DOI:** 10.3389/fmicb.2024.1314528

**Published:** 2024-02-20

**Authors:** Wei Liu, Zhongxia Li, Xiaolei Ze, Chaoming Deng, Shunfu Xu, Feng Ye

**Affiliations:** ^1^Institute of Plant Protection and Microbiology, Zhejiang Academy of Agricultural Sciences, Hangzhou, China; ^2^BYHEALTH Institute of Nutrition and Health, Guangzhou, China; ^3^Department of Gastroenterology, The First Affiliated Hospital of Nanjing Medical University, Nanjing, China

**Keywords:** Bile acids, gut microbiota, metabolism, probiotics, *in vitro* fermentation model

## Abstract

The consumption of probiotics has been extensively employed for the management or prevention of gastrointestinal disorders by modifying the gut microbiota and changing metabolites. Nevertheless, the probiotic-mediated regulation of host metabolism through the metabolism of bile acids (BAs) remains inadequately comprehended. The gut-liver axis has received more attention in recent years due to its association with BA metabolism. The objective of this research was to examine the changes in BAs and gut microbiota using an *in vitro* fermentation model. The metabolism and regulation of gut microbiota by commercial probiotics complex containing various species such as *Lactobacillus, Bifidobacterium*, and *Streptococcus* were investigated. The findings indicated that the probiotic strains had produced diverse metabolic profiles of BAs. The probiotics mixture demonstrated the greatest capacity for Bile salt hydrolase (BSH) deconjugation and 7α-dehydroxylation, leading to a significant elevation in the concentrations of Chenodeoxycholic acid, Deoxycholic acidcholic acid, and hyocholic acid in humans. In addition, the probiotic mixtures have the potential to regulate the microbiome of the human intestines, resulting in a reduction of isobutyric acid, isovaleric acid, hydrogen sulfide, and ammonia. The probiotics complex intervention group showed a significant increase in the quantities of *Lactobacillus* and *Bifidobacterium* strains, in comparison to the control group. Hence, the use of probiotics complex to alter gut bacteria and enhance the conversion of BAs could be a promising approach to mitigate metabolic disorders in individuals.

## Introduction

The human gut provides a dynamic habitat for microbial communities. In normal bodily conditions, external nourishment and internal substances enter the digestive system to supply sufficient materials for the host and its microbiota. Bile acids (BAs) are many endogenous amphiphilic molecules combined with taurine or glycine. Bile acids are synthesized in the liver through a complex enzymatic reaction, which is catalyzed by cholesterol (Singh et al., 2019).

While eating, the gallbladder releases bile salt into the intestine to aid in the digestion of nutrients from food, which is constantly present in the intestine along with chyme. Eventually, it is absorbed again at the ileum's conclusion and sent back to the pool of bile salts via circulation. Around 95% of bile salts are efficiently absorbed again during the process of intestinal liver circulation. During the process, only a limited quantity of bile salts is permitted to be released into the large intestine, where they engage in the metabolic transformation process of gut microbiota (Trauner and Boyer, 2003).

Maintaining an optimal composition within the bile acid pool and metabolic homeostasis is heavily influenced by the interaction between the gut and liver. These mechanisms may contribute to the hindrance of high blood sugar, abnormal blood lipid levels, excessive body weight, and the development of diabetes. BAs, which are generated by the host and altered by gut microbiota, serve as a crucial factor in the absorption of nutrients, transmission of hormonal signals, control of lipids and cholesterol, inflammation, and maintenance of energy balance (Tian et al., 2020).

The liver initiates the production of BA through the synthesis of cholesterol. The majority of BAs are taken in by the intestine through the hepatic portal vein and subsequently return to the liver to initiate the cycle once more. Rodents have primary bile acids including cholic acid (CA), chenodeoxycholic acid (CDCA), and α- and β-muricholic acids, as well as secondary BAs such as murideoxycholic acid (MDCA), hyodeoxycholic acid (HDCA), and ω-Muricholic acid (ω-MCA) (De Aguiar et al., 2013). In comparison to other mammals, the liver of humans generates two main bile acids, namely CA and CDCA, while the bile acid pool contains secondary BAs including deoxycholic acid (DCA) and lithocholic acid (LCA). CA and CDCA are significant biological agents in the human body (Li et al., 2020). In the gut, the primary bile acids undergo a conversion process to become secondary bile acids during digestion. Apart from being characterized as primary or secondary BAs, each BA can be conjugated or unconjugated (Puri et al., 2018). Furthermore, the gut microbiome plays a crucial role in the breakdown of BAs. Bacteria that metabolize BAs, especially gut commensals that catalyze the dehydroxylation of primary BAs into7-dehydroxylation BA, offer a hopeful opportunity to regulate the BAs reservoir and thereby influence the physiology of the host (Marion et al., 2019).

Nevertheless, in the case of humans, the buildup of BAs has been linked to harm to the liver, long-term liver illness, inflammation, and the development of tumors (Zhou and Hylemon, 2014). Elevated concentrations of secondary BAs in both feces and blood have been linked to the development of cholesterol gallstones and colon cancer (Li et al., 2019). In addition to cancer, the disruption of the intestinal microecological balance is connected to the majority of chronic illnesses in humans, encompassing conditions linked to inflammation, metabolism, heart health, immune system, nervous system, and mental health. The majority of symbiotic microorganisms inhabit the human intestine directly, and alterations in the equilibrium of the population are the main cause of intestinal diseases. As a result, there is a growing emphasis on the management of gut bacteria to prevent and treat specific illnesses (Baker et al., 2011).

Intestinal health is significantly influenced by the interaction of probiotics and BAs, as indicated by recent research. The intestinal microbiota transforms conjugated bile acids into unconjugated bile acids through the action of bacterial bile salt hydrolase (BSH). These unconjugated bile acids are then subjected to various modifications such as 7-dehydroxylation or 12-dehydroxylation, amidation, oxidation-reduction, esterification, and desulfation, resulting in the formation of secondary bile acids. For example, *Bacteroides intestinalis* has been recognized for transforming primary BAs into secondary BAs, which could potentially cause cancerous effects, through the process of deconjugation and dehydration (Fukiya et al., 2009). *Lactobacillus delbrueckii* subsp. *bulgaricus* (LDB) has been shown its ability to actively acquire BAs through various mechanisms, such as transportation driven by the bacterial transmembrane proton gradient and binding facilitated by bacterial S-layer proteins (Hou et al., 2020). Furthermore, the study revealed that *Lactobacillus rhamnosus* GG (LGG) not only suppressed the synthesis of hepatic BAs but also increased their secretion by activating the FXR/FGF15 signaling pathway and controlling the deconjugation of primary BAs mediated by gut microbiota. In mice, these impacts hinder the occurrence of excessive liver damage and fibrosis caused by BAs (Liu et al., 2020b). It was noted that the administration of VSL#3, a blend of probiotics, resulted in elevated excretion and detachment levels of fecal BAs in mice. Additionally, there was an increase in the production of liver BAs and alterations in the gut microbiota of the mice (Degirolamo et al., 2014).

Currently, there is a wealth of data on the tolerance of probiotics to BAs, but little information exists regarding the impact of probiotic metabolism on BAs. The present study aimed to examine the alterations in structure and levels of short-chain fatty acids (SCFAs) in a probiotic complex following a 24-hour fermentation period. In an *in vitro* fermentation model, the metabolism of individual probiotics and a mixture of probiotics were investigated both before and after fermentation. This research aims to clarify the connection between probiotics and BAs metabolism, indicating that probiotics with the ability to regulate BAs could be used as an alternative approach to enhance liver metabolism.

## Materials and methods

### Probiotic complex and reagents

Mixed probiotic products tested in this study were provided by BYHEALTH Co., LTD, namely Yep. There are four strains in the probiotics complex: *L. acidophilus* DDS-1 (9 × 10^9^ CFU/g), *L. rhamnosus* UALR-06 (4 × 10^9^ CFU/g), *B. lactis* UABIA-12 (3.5 × 10^9^ CFU/g), *B. longum* UABI-14 (5 × 10^8^ CFU/g). The complex probiotics were used to evaluate the effect on gut microbiota and the ability to metabolize BAs. All of the standard bile acids were purchased from CNW (Shanghai, China) and IsoReag (Shanghai, China). Before analysis, the stock solutions were diluted using methanol (MeOH) to create working solutions.

### Stool sample collection

Samples of fresh feces from 20 individuals in good health, consisting of 10 males and 10 females, were collected. Every volunteer had to adhere to their regular diet and had no previous or current use of antibiotics, probiotics, or any other supplementary medications in the past 3 months. Prior to sample collection for this study, these volunteers, who are free from any intestinal diseases, provided their informed consent. The specimens were kept at a temperature of 4°C and were handled within a time frame of 4 h after being gathered.

### Anaerobic fermentation broth preparation

The necessary culture medium was formulated based on previously published literature (Liu et al., 2020a). In brief, Yeast extract-Casein hydrolysate-Fatty Acids (YCFA) and MRS-L (Man-Rogosa-Sharpe with 1.5% L-cysteine) media as the fermentation medium was aliquoted into 5 mL bottles using a peristaltic pump under anaerobic conditions through nitrogen filling and then sealed with a rubber plug. The culture media were autoclaved before use. Individual probiotics were grown in MRS-L medium.

### Batch *in vitro* fermentation

One gram of fecal samples was weighed into centrifuge tubes and diluted in 10 ml of phosphate buffer solution (PBS). To achieve thorough blending, the samples underwent oscillation on a vortex shaker and were subsequently filtered twice using a double-layer sterile filter screen to eliminate any insoluble large particles. Afterward, a fecal suspension with a concentration of 10% was prepared using PBS to serve as the initial stock solution for the gut microbiota.

A YCFA medium was prepared according to a method reported in the literature (Tidjani Alou et al., 2020), and the final pH of the medium was adjusted to 6.8 ± 0.1. On an ultra-clean workbench, a sterile syringe was used to inoculate YCFA basic medium with 500 μL of fecal suspension in PBS. Next, the probiotics complex was inoculated with 1% (w/w) solution and injected into the anaerobic fermentation vial. Samples not inoculated with probiotics served as the blank control (CK). All anaerobic fermentation vials were placed inside the anaerobic workstation under an 85% N_2_/10% H_2_/ 5% CO_2_ Atmosphere to undergo fermentation at 37°C for 24 h. The experimental scheme of fermentation *in vitro* is shown in Supplementary Figure 1.

### BAs sample preparation and detection

Fresh BAs from cattle and pig were purchased from farmhouse local specialty store in Changsha. Human bile samples were obtained from patients who had undergone endoscopic retrograde cholangiopancreatography (ERCP) procedures within the Department of Gastroenterology at the First Affiliated Hospital of Nanjing Medical University. Bile aspirations were acquired subsequent to the cannulation of the papilla, facilitated by the insertion of a sterile 7 French standard endoscopic retrograde cholangiography catheter into the common bile duct (Cook, Ireland), followed by the extraction of bile into a sterile 20 mL syringe. The study protocol and informed consent form were approved by the ethics committee of the First Affiliated Hospital of Nanjing Medical University (2024-SR-037). A total of three types of BAs derived from cattle, pigs, and humans were collected. The pig group contained five samples each, while the human group provided six samples. The control and treatment groups were subjected to the BAs assay in the *in vitro* fermentation model. In the control group, there was 50 μL of human bile mixed with 5 mL of MRS-L medium, whereas in the treatment group, there was a mixture of 50 μL of overnight bacterial suspension, 50 μL of human bile, and 5 mL of MRS-L medium. BA experiment was done in triplicate for each sample. Furthermore, a volume of 1 mL from suspensions co-cultured for 24 h was obtained and then centrifuged at 10,000×g for 3 min. Subsequently, 500 μL of the resultant supernatant was utilized for BA detection.

The quantification of the BAs was performed using the ACQUITY UPLC-Xevo TQ-S system (Waters Corp., USA), which combines ultra-performance liquid chromatography with tandem mass spectrometry (UPLC-MS/MS). The cryopreserved BAs were first removed and dissolved on ice before being pipetted into 5 μL samples of BAs with 15 μL pure water.

The suspensions were combined in a tube for centrifugation, followed by the addition of 180 μL of the solvent containing the internal standard acetonitrile (ACN)/MeOH = 80/20 (v/v). To ensure sufficient protein precipitation, the samples were homogenized at a speed of 1,450 × g at 10°C, followed by freezing storage at −20°C. Afterward, the specimens were spun at 13,500 × g at a temperature of 4°C for 20 min.

### Determination of SCFA and gases of fermentation products

The analysis of SCFAs was conducted utilizing a Shimadzu GC2010 gas chromatograph (GC) manufactured by Shimadzu Corporation in Kyoto, Japan. For the analysis of SCFAs, the supernatant was removed. Initially, 500 μL supernatant was combined with 100 μL crotonic acid / metaphosphoric acid, thoroughly mixed, and then placed in a freezer at −30°C for 24 h to complete the acidification of the samples. Samples treated with acid were subjected to centrifugation at 4°C using a speed of 10,000×g for 3 min. at 4°C. Following passage through a 0.22 μm millipore filter, an injection of 10 μL of the supernatant was made using an Agilent DB-FFAP column (0.32 mm×30 m×0.5 μm; Agilent, USA). The nitrogen flow rate as the carrier gas remained constant at 12 mL/min while the split ratio was adjusted to 8.0. The detector was supplied with hydrogen, air, and tail at flow rates of 40 mL/min, 400 mL/min, and 30 mL/min, respectively. GC was conducted using a temperature gradient that started at 80°C and increased to 190°C at a rate of 10°C per minute. After reaching 240°C at a speed of 40°C/min for 5 min, it was then kept constant for an extra 0.5 min. The FID and gasification chamber were both set to 240°C. Following a 24-h period, the fermentation vials were assessed for gas production (CO_2_, CH_4_, H_2_, H_2_S, and NH_3_) using an APES-BC5-B fermentation gas analyzer manufactured by Empaer Technology Co., Ltd. (located in Shenzhen, China) and equipped with five highly responsive gas transducers.

### Sequencing the gut microbiota using 16s rRNA

To remove the liquid above, the 24-h fermentation mixture was spun at a speed of 9000×g for 3 min. DNA extraction and analysis of 16S rRNA sequencing were performed on the pellet. After the completion of the experiment, the DNA samples obtained were measured using agarose gel electrophoresis and a microplate reader called Nanodrop 2000. The DNA amplification technique was used to obtain the v3-v4 region of the 16sRNA. Subsequently, PCR primers were designed to flank the v3-v4 region of the bacterial 16S rDNA, with primer 343F (5'-tacggraggcagcag-3') and primer 798R (5'-agggtatctaatcct-3') bands being utilized.

The PCR amplification conditions were as follows: initial denaturation at 94°C for 5 min, followed by seven cycles of denaturation at 94°C for 30 s, annealing at 56°C for 30 s, extension at 72°C for 20 s, another extension at 72°C for 5 min, and a final hold at 4°C. Agarose gel electrophoresis was employed to analyze the PCR products, followed by purification of the amplification products using magnetic bead purification and quantification using Qubit. Libraries were constructed using a Bioanalyzer (Aglient 2100).

The original double-ended sequence was generated by sequencing Illumina MiSeq and then de-hybridized using Trimmomatic software. The full sequence was obtained by splicing the double-ended sequence using Flash software (version 1.2.11) after complete generation. To eliminate sequences with N bases, single base repetition times >8, and insufficient length, the split_libraries (version 1.8.0) software in QIIME was employed for spliced sequences. Afterward, the UCHIME program (2.4.2 version) was employed to eliminate chimeras from the sequences, acquiring top-notch sequences for the division of Operational Taxonomic Units (OTU) in the following steps. The National Center for Biotechnology Information Short Read Archive received all human consensus sequencing data with accession no. PRJNA1003221.

### Statistical analysis

Graph Pad Prism8 and IBM SPSS Statistics 25 were used to statistically analyze the experimental data, which were then presented as the mean ± SD. The data with normal distribution underwent a comparative analysis among multiple groups using the Friedman M non-parametric test, considering the Bonferroni corrected significance value for multiple tests. The results of the test for homogeneity of variance and their statistical significance were indicated by the *p*-value (^*^p < 0.05, ^**^p < 0.01, ^***^p < 0.001). Significantly regulated metabolites of BAs between groups were determined by VIP and absolute Log_2_FC (fold change). VIP values were extracted from OPLS-DA result, which also contains score plots and permutation plots, and were generated using R package MetaboAnalystR.

Using the Vsearch software (version 2.4.2), the high-quality sequences obtained were categorized into OTUs with a similarity of at least 97%. The representative sequence of each OTU was determined by selecting the most abundant sequence through the QIIME software. The OTU annotation information was obtained by comparing the representative sequences with the database using the Naive Bayesian classification algorithm of the RDP classifier. To build the phylogenetic tree, we utilized the Pynast (v0.1) program for constructing the phylogenetic relationship of representative sequences of OTUs and obtaining the resulting tree.

## Results

### Characteristics of BAs

**Figure 1:**
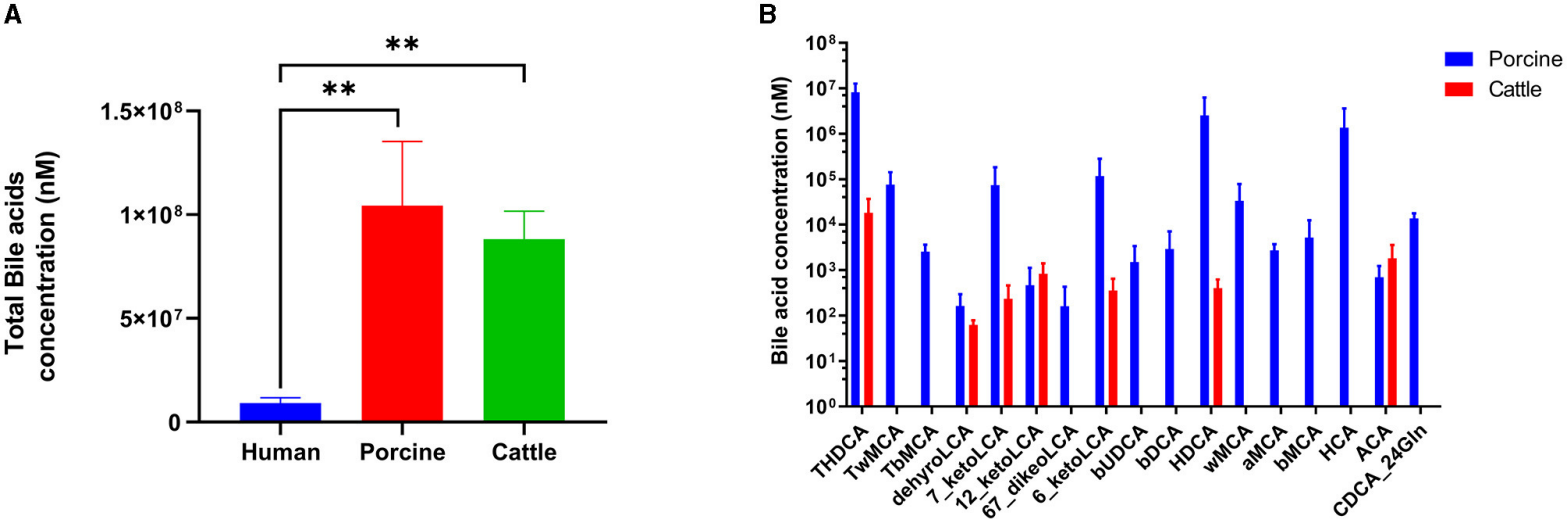
Bile acid profiles in humans, pigs, and cattle. **(A)** Content of total bile acid. **(B)** Unique bile acids in pigs and cattle. Significance was determined as *p < 0.05 (significant), **p < 0.01 (very significant) using one-way ANOVA (*Post-hoc* Dunnett's test).

Utra-performance liquid chromatography coupled with triple quadrupole mass spectrometry (UPLC–TQMS) was used to collect and analyze five fecal samples obtained from pig, cattle, and human sources. Figure 1 illustrates that the three distinct host samples exhibited variations in both the content and types of total bile acids (TBAs). Specifically, when comparing the BAs of pigs and cows, the levels of TBAs in humans, pigs, and cattle were in a ratio of 1:11:10. Previous studies have demonstrated that the TBAs of pigs and cattle are roughly 11 and 10 times greater than those of humans, respectively. The TBAs of pigs were most abundant, followed by cattle and humans. In pigs, TωMCA, TβMCA, βUDCA, βDCA, ωMCA, HCA, αMCA, βMCA, and CDCA-24Gln stood out as distinct from the BAs species found in cattle.

### Bacterial alteration of BA profiles

**Figure 2:**
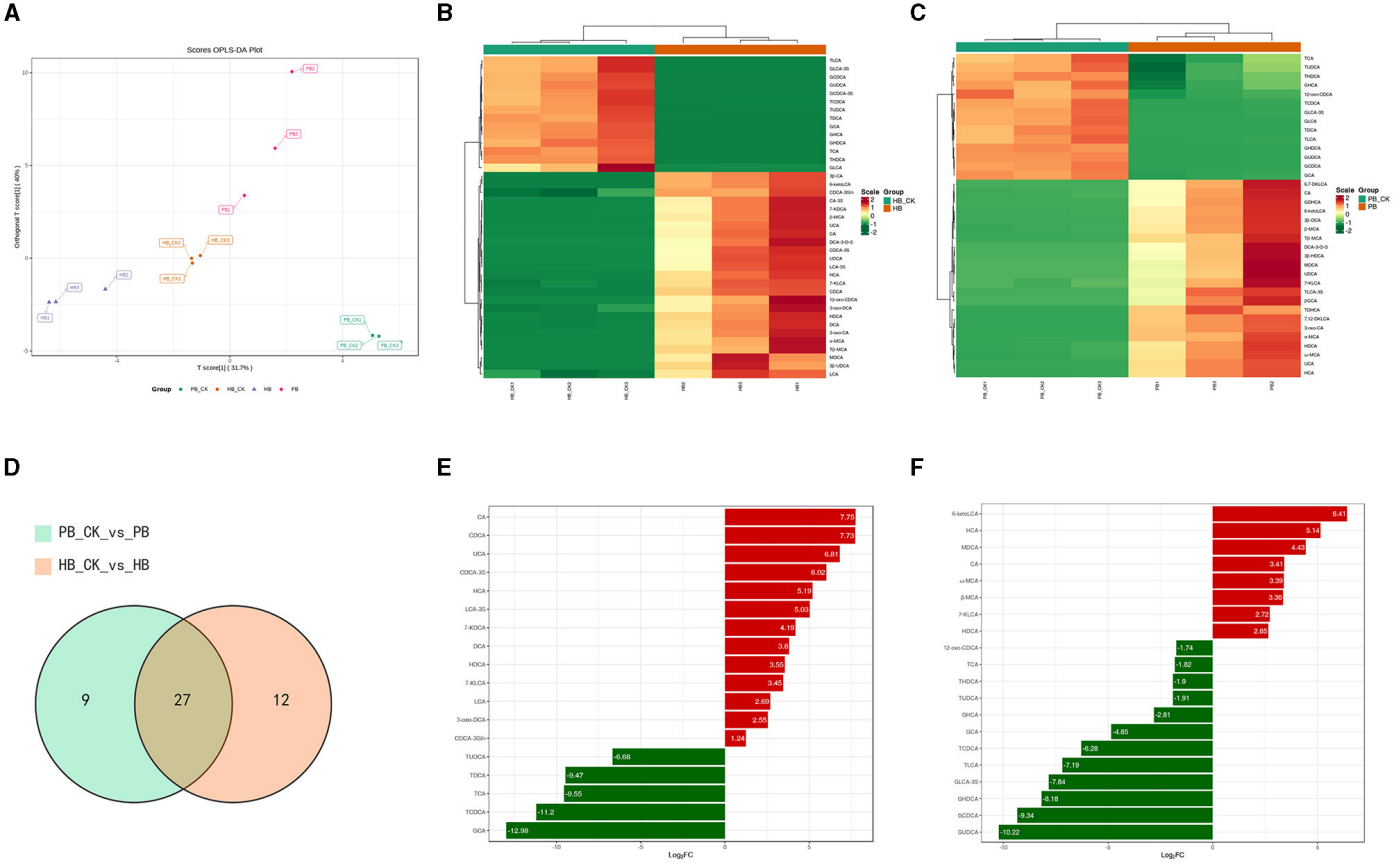
Metabolism characterization of bile acids against complex probiotics in pigs and humans. **(A)** Orthogonal partial least squares discriminant analysis (OPLS-DA) of BA in the human (HB) and pig groups (PB). **(B)** Heatmap of BAs metabolism in humans. **(C)** Heatmap of BAs metabolism in pig. The red color shows an increased expression level while the blue color displays decreased expression level. Hierarchical classification was performed to group metabolites from each group. Dendrograms show clustering between metabolites which were closely related based on their estimated Euclidean distance. **(D)** Venn diagram comparison analysis of BA metabolism in the presence of probiotics. **(E)** up-regulated BA levels in humans compared with the control group. **(F)** down-regulated BA levels in pigs compared with the control group.

To explore the disparity in BA metabolism between pigs and humans, we analyzed the range of BAs in a laboratory-based fermentation model. To evaluate the level of expression, a system of color coding was used, which involved the utilization of red and blue. Red was used to indicate a higher level of expression, while blue was used to represent a lower level of expression. White was used to signify no change. After 24 h of fermentation with probiotics, Figure 2A clearly distinguishes the metabolic variety of human BAs from that of porcine BAs. The OPLSDA model was used to assess the diversity in the metabolite composition of BA varieties. The evaluation parameters of the model, R^2^ = 0.97 and Q^2^ = 0.939, demonstrated the reliability and validity of the OPLSDA model. Within the porcine category, there was a notable decrease in 14 BA species and a significant increase in 22 BA species. Similarly, the human BA group experienced a significant decrease in 14 BA species and a noteworthy increase in 25 BA species. Regarding the metabolism of BAs, both of them exhibit a comparable pattern of change, where secondary BAs undergo modification through conjugation with either glycine (forming glyco-conjugated bile acids) or taurine (forming tauro-conjugated bile acids) during probiotic fermentation (as shown in Figures 2B, 2C). In Figure 2D, a total of 27 BA species were found to be present in both the metabolism of human BA and porcine BA. Among these metabolites, the concentrations of CA, HCA, and HDCA exhibited a significant increase, while the levels of GCA, TCDCA, and TUDCA showed a substantial decrease in the top 20 metabolites with the highest fold change when probiotics were present (as shown in Figures 2E, F, Supplementary Tables 1, 2). Complex probiotics Yep had varied outcomes for different derived BAs. The findings may suggest that these characteristics of probiotics possess a strong capacity to alter the metabolism of BAs, rendering them promising candidates for interventions aimed at enhancing wellbeing.

### Effects of probiotics complex on SCFAs and intestinal gases

**Figure 3:**
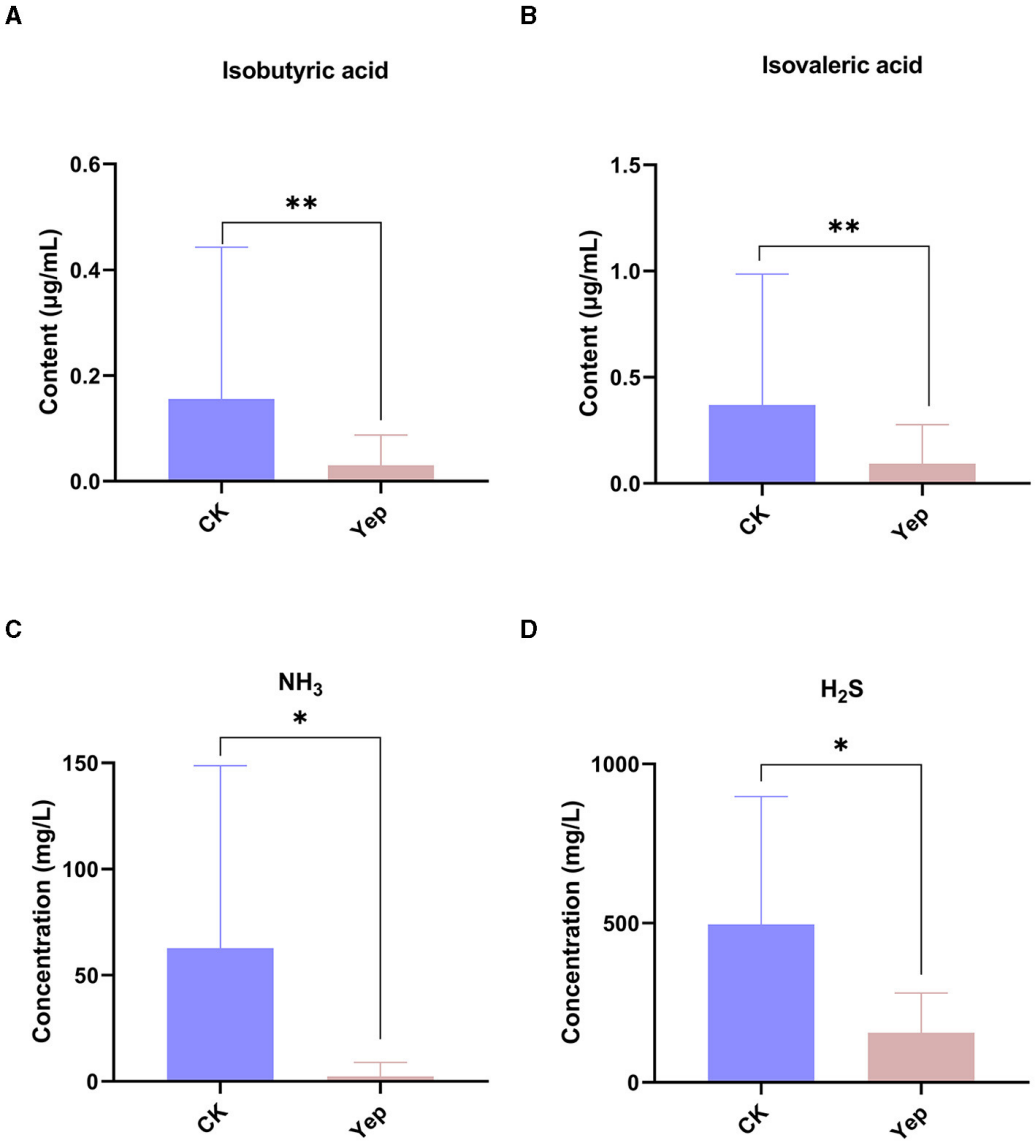
The levels of short-chain fatty acid and gases produced by gut microbiota after 24-h fermentation processes under the probiotics complexes intervention. CK represents the control group, Yep represents the probiotics complex, *p < 0.05, significant. **p < 0.01, very significant. ***p < 0.001, extremely significant.

The determination involved SCFAs such as acetic acid, propionic acid, isobutyric acid, butyric acid, isovaleric acid, and valeric acid, as depicted in Figure 3. The levels of isobutyric acid (*p* = 0.009) and isovaleric acid (*p* = 0.002) in the complex probiotics Yep were notably reduced when compared to the CK group. In comparison to the CK group, the complex probiotics Yep exhibited a notable reduction in the concentration of NH_3_.

### Effects of probiotics complex on gut microbiota

**Figure 4:**
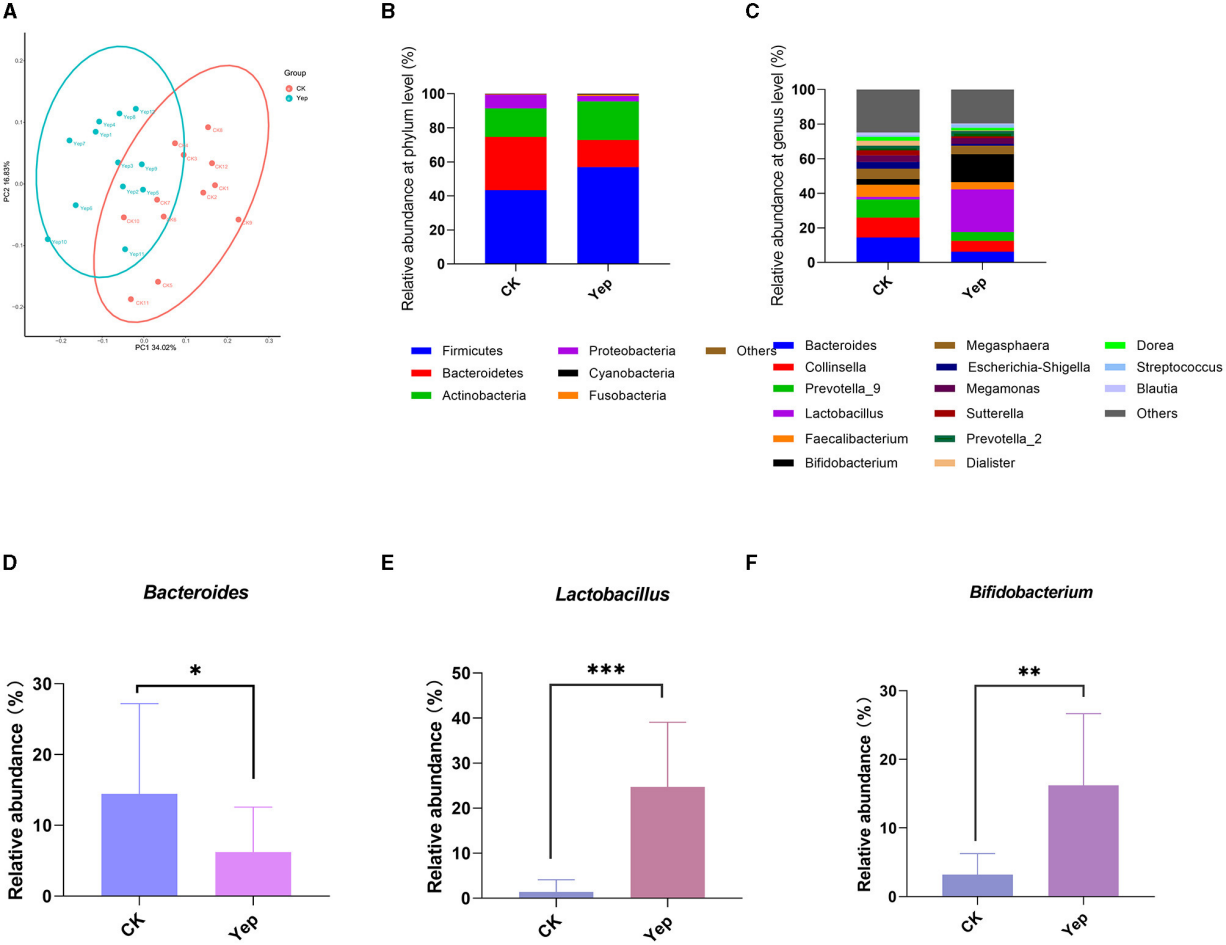
Effect of complex probiotics on gut microbiota in humans. **(A)** PCoA analysis based on weighted unifrac distances. **(B)** The profiles of human gut microbiota on phylum level after the probiotics complex intervention. **(C)** The profiles of human gut microbiota on genus level after the probiotics complex intervention. **(D–F)** Relative abundance of *Bacteroides, Lactobacillus*, and *Bifidobacterium* in different groups. Compared between the CK and Yep group; significance was determined as *p < 0.05 (significant), **p < 0.01 (very significant) using Student's t-test.

The PCoA analysis based on the plot of the weighted unifrac distance matrix showed no apparent cluster differentiation between the CK group and the probiotics complex. On the other hand, the probiotics mixture was far apart from the rest of the groups and exhibited a greater level of variation. The data indicate that the probiotics complex caused noticeable alterations in the gut microbiota (Figure 4). After 24 h of fermentation, *Firmicutes, Bacteroidetes, Actinobacteria*, and *Proteobacteria* were the predominant gut microbiota in each group at the phylum level. Furthermore, there were slight alterations noticed in the proportion of *Firmicutes* and *Bacteroidetes*. Specifically, when compared to the other groups, complex probiotics Yep notably enhanced both this ratio and the ratio of *Actinobacteria* simultaneously.

At the level of genus, the probiotic mixture changed the gut microbiota and notably boosted the presence of *Bacteroides, Lactobacillus*, and *Bifidobacterium* genera in comparison to the control group (Figure 4). Furthermore, the complex group exhibited significantly lower levels of various opportunistic pathogens, such as *Escherichia-Shigella* and *Sutterella* genera, in comparison to the CK group.

### Correlation between SCFAs and gas production

**Figure 5:**
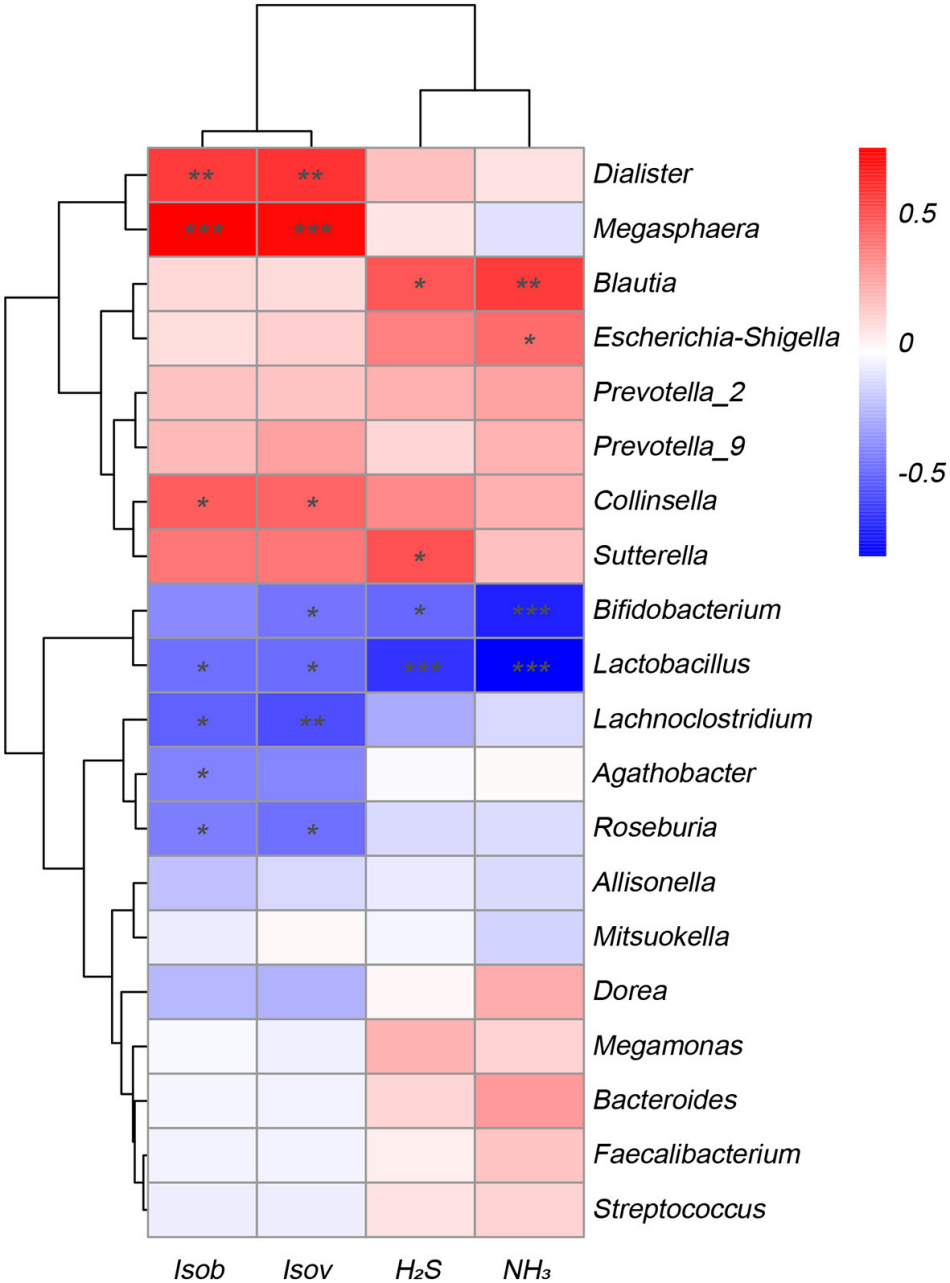
Correlation analysis of fecal microbiota at the genus level with metabolites SCFAs and gases in the Yep-treated samples. Isob, isobutyric acid; Isov, isovaleric acid; The correlation heatmap was measured using the Spearman correlation coefficient. *0.01 < *p* ≤ 0.05; **0.001 < *p* ≤ 0.01; ***p ≤ 0.001.

To explore the correlation between the microbiota involved in fermentation and the resulting SCFAs and gases, this study conducted a correlation analysis between the top 20 various bacterial genera with the greatest abundance and fermentation metabolites. Figure 5 displayed that various bacteria exhibited distinct associations with the production of gases and SCFAs. The amounts of isobutyric acid and isovaleric acid exhibited an inverse relationship with the genera *Lactobacillus, Lachnoclostridium*, and *Roseburia*, while displaying a positive correlation with the genera *Dialister, Megasphaera*, and *Collinsella*. Similarly, the levels of NH_3_ and H_2_S synthesis exhibited an inverse relationship with the *Bifidobacterium* and *Lactobacillus* species, and they displayed a positive correlation with the *Blautia* species. Conversely, *Escherichia*-*Shigella* showed a negative correlation with NH_3_.

## Discussion

Bile consists of compounds that are produced by the liver, gallbladder, and bile duct cells. These compounds can be either endogenous or exogenous, reflecting both typical and atypical metabolic processes of these organs. Studying the composition of bile can offer valuable insights into biomarkers of hepatobiliary diseases, aiding in their diagnosis and therapeutic utilization.

Research has shown that BAs have a vital function in maintaining balance in the liver, gallbladder, intestines, and aiding in the process of digestion. Moreover, the intestinal flora greatly affects the variety of bile acid collections by breaking down conjugates and removing hydroxyl groups or changing their configuration, which has important consequences for the host. As a major contributor to BAs metabolism, BSH produces secondary BAs. Of the many gut microbiota, *Lactobacilli* and *Bifidobacteria* have been most frequently studied for BSH activity.

Nevertheless, research is scarce on the correlation between BAs derived from humans and certain probiotics. The metabolism of BAs and the changes in gut microbiota were investigated in this study using an *in vitro* fermentation model and by introducing external probiotics for intervention. Typically, the *in vitro* BAs assay models solely consist of pigs, cattle, or mice, and BAs were employed to assess the resistance and tolerance of probiotic strains, disregarding the definitive host. In contrast to BAs found in pigs and cows, the composition of BAs in humans is completely different (Begley et al., 2005), whereby the concentration of total BAs in pig and cow bile is ~10 times greater than that in human bile. Moreover, selected BAs are unique in pigs compared with cattle bile, such as TβMCA, TωMCA, etc. This suggests that pig BA models can be utilized to evaluate and forecast the efficacy of various interventions on BA metabolism, particularly in the bile of humans.

Intestinal BAs have the potential to impact the composition of the gut microbiota, subsequently exerting an influence on it. Deconjugation is the process by which the conjugated BAs, like CA and CDCA, are metabolized and ultimately converted into secondary BAs, including DCA, HDCA, HCA, or THDCA. The BA profiles underwent significant changes over time following the introduction of probiotics complex Yep. In a previous study by Bansal et al. (2020) it was found that DCA, which is a microbial metabolic byproduct obtained from secondary bile acids, effectively decreased the colonization and inflammation induced by *C. perfringens* in necrotic enteritis among chickens. Several BAs, such as chenodeoxycholic, lithocholic, and ursodeoxycholic acids, were discovered to hinder spore germination in a separate investigation (Davis et al., 2016). Furthermore, bacteria possessing BSH activity are responsible for the deconjugation of BAs. In previous studies, *Lactobacilli, Clostridium, Bacteroides*, and *Bifidobacteria* were found to possess functional BSH (Jones et al., 2008).

The regulation of BAs is governed by a wide range of nuclear and membrane receptors. Wan and Sheng (2018) identified FXR and TGR5 as the main representatives of nuclear and membrane receptors, respectively. Activation of the FXR and TGR5 receptors by CDCA, DCA, CA, and LCA regulates various host processes, such as energy metabolism, reduction of hepatic BAs burden, and alleviation of toxic cellular damage induced by BAs in cholestasis. Additionally, these receptors also influence glucose, lipids, and anti-inflammatory reactions (Gonzalez et al., 2015). Moreover, the TGR5 receptor gets activated by the LCA, DCA, TLCA, and TCDCA bile acids. According to recent research, it has been found that the TGR5 ligand reduces the production of inflammatory cytokines when exposed to lipopolysaccharides (LPS) (Hogenauer et al., 2014). The HCA types serve as new indicators for metabolic disorders and enhance the regulation of glucose balance by means of a unique TGR5 and FXR communication pathway, resulting in potential benefits for diabetes prevention (Zheng et al., 2021). It was reported that the control of THDCA levels has been found to regulate rheumatoid arthritis in a persistent cold climate (Liu et al., 2023). Inhibiting the PPARα nucleus cytoplasm pathway, the diet supplemented with HDCA was separately analyzed and found to improve non-alcoholic fatty liver disease (Kuang et al., 2023). Consistent with this observation, we noticed a significant increase in the levels of CDCA, DCA, CA, and HCA in this study when the probiotics complex Yep was administered.

Bile acids (BAs), suggested as a possible indicator of liver damage, have been under consideration for many years. Elevated concentrations of linked BAs in cirrhosis or chronic hepatitis may serve as useful markers of liver impairment for hepatocellular carcinoma in individuals with liver cirrhosis. Elevated serum levels of TCDCA, TUDCA, GCA, TCA, and GCDCA were found in women suffering from severe intrahepatic cholestasis of pregnancy, as indicated by a study (Ovadia et al., 2019). The presence of alterations in BAs is closely connected to the pathological changes that occur during the advancement of cirrhosis. The findings of this research were indistinguishable from those of another study (Liu et al., 2019a). Individuals diagnosed with early cirrhosis showed a significant rise in the levels of overall BAs, such as GCA, GCDCA, TCA, TCDCA, and TUDCA, compared to those diagnosed with chronic hepatitis. Furthermore, these increased levels were discovered to have a strong correlation with the identification of cirrhosis. Moreover, these elevated concentrations were found to be closely linked to the diagnosis of cirrhosis. There were notable associations between the fibrosis stages and TCDCA, GCDCA, GCA, and TCA, indicating positive correlations. Higher concentrations of GCDCA, TCDCA, GCA, and TCA exhibited a positive correlation with the advancement of alcoholic liver disease in individuals (Sugita et al., 2015). Additionally, it was hypothesized that GCDCA, GCA, and GUDCA could serve as more reliable indicators of abstaining from alcohol (Wang et al., 2020). The patients with alcoholic cirrhosis showed a significant rise in the levels of conjugated primary bile acid metabolites (GCDCA, TCDCA, GCA, TCA) compared to secondary bile acid metabolites (except for glycohyocholate), indicating a correlation between disease severity and the progressive increase of these subsets. Furthermore, mice that were consistently fed a high-fat diet (HFD) also exhibited the spontaneous formation of liver tumors, accompanied by a notable rise in levels of bile acids within the liver. The probiotics complex intervention in humans resulted in a significant decrease in the levels of TUDCA, TDCA, TCA, TCDCA, and GCA, which was quite fascinating. The BAs metabolism is believed to be improved by the physiological activities of the probiotics complex Yep, which are considered beneficial for human health in this study.

In the present study, the administration of the probiotics complex decreased isobutyric acid and isovaleric acid levels. These branched short-chain fatty acids are derived from the degradation of amino acids valine, leucine, or isoleucine and have been shown to enhance insulin-stimulated glucose uptake and improve insulin sensitivity (Qu et al., 2017). Consequently, the findings potentially imply a transition from a carbohydrate-based to a protein-based fermentation milieu, thereby indicating alterations in the microbial composition (Liu et al., 2019b). In addition, the isobutyric acid and isovaleric acid levels were correlated negatively with *Lactobacillus, Lachnoclostridium*, and *Roseburia*, while correlated positively with *Dialister, Megasphaera*, and *Collinsella*. Similarly, the concentrations of H_2_S and NH_3_ exhibited a reduction after fermentation, indicating that the probiotics complex exerts a modulatory influence on gut microbiota, resulting in a decrease in the abundance of detrimental bacteria associated with gas production. Importantly, a negative association was found between H_2_S and NH_3_ and the genera *Bifidobacterium* and *Lactobacillus*, while a positive association was observed with the genus *Blautia*, which has been linked to inflammation (Maeda and Takeda, 2017).

A symbiotic gut microbiota consists of numerous bacteria and metabolites expressed by bacteria and the host, and they can regulate the microecological balance of the intestinal tract, protect intestinal mucosa, and decrease inflammation while enhancing the metabolism of lipids. Compared with the discoveries in existing literature, there are some novelties in our study. This study represents the first attempt to compare the variations in BAs between human, pig, and cattle species. Additionally, comparison studies *in vitro* on BAs and multi-species probiotics in humans and pigs have never been investigated. However, it is imperative to recognize that the existing research is still in its nascent phases and poses numerous unresolved enigmas and obstacles. In our study, only two independent assessments to determine the effects of the probiotics complex on BAs and gut microbiota were conducted, we didn't account for the potential impact of the simultaneous presence of both factors on microbial metabolism, which serves as a limitation of our study. To enhance comprehension of the probiotics' mechanism of action, further inquiries are imperative, encompassing supplementary clinical trials, identification of bioactive constituents, and optimization studies to treatment dosages and durations. Moreover, comprehensive evaluations of the interactions and safety of BAs and probiotics are also indispensable to provide additional avenues and alternatives for the treatment of liver ailments.

## Conclusion

The current research found that the complex probiotics Yep had an impact on the microbiota in the human gut. It led to an increase in the prevalence of *Bacteroides, Lactobacillus*, and *Bifidobacterium*, while reducing the prevalence of the opportunistic pathogens *Escherichia-Shigella* and *Sutterella*. At the same time, the intricate probiotics regulated the metabolism of BAs and intestinal gases. The present findings indicate the possibility of using probiotic intervention to enhance gut microbiota and support gastrointestinal wellbeing.

## Data availability statement

The datasets presented in this study can be found in online repositories. The names of the repository/repositories and accession number(s) can be found at: https://www.ncbi.nlm.nih.gov/, PRJNA1003221.

## Ethics statement

Ethical approval was attained by the ethics committee of the First Affiliated Hospital of Nanjing Medical University (2024-SR-037). Ethical approval was not required for the studies involving animals in accordance with the local legislation and institutional requirements because The bile acids from porcine and cattle were commercially available and used as purchased. Written informed consent was obtained from the owners for the participation of their animals in this study.

## Author contributions

WL: Writing—original draft, Writing—review & editing, Conceptualization, Funding acquisition. ZL: Writing—original draft, Writing— review & editing, Conceptualization. XZ: Writing—review & editing, Formal analysis. CD: Writing—review & editing, Formal analysis. SX: Writing—review & editing, Formal analysis. FY: Writing—review & editing, Supervision, Conceptualization, Formal analysis, Resources.
