# Supplementary material for: Multispecies probiotics complex improves bile acids and gut microbiota metabolism status in an in vitro fermentation model
Source: Front Microbiol. 2024 Feb 20;15:1314528. doi: 10.3389/fmicb.2024.1314528 (PMC10913090; doi:10.3389/fmicb.2024.1314528)

Supplementary figure 1.The experimental scheme of the probiotic complex Yep intervention by fermentation in vitro model in presence of human gut microbiota and bile acids.

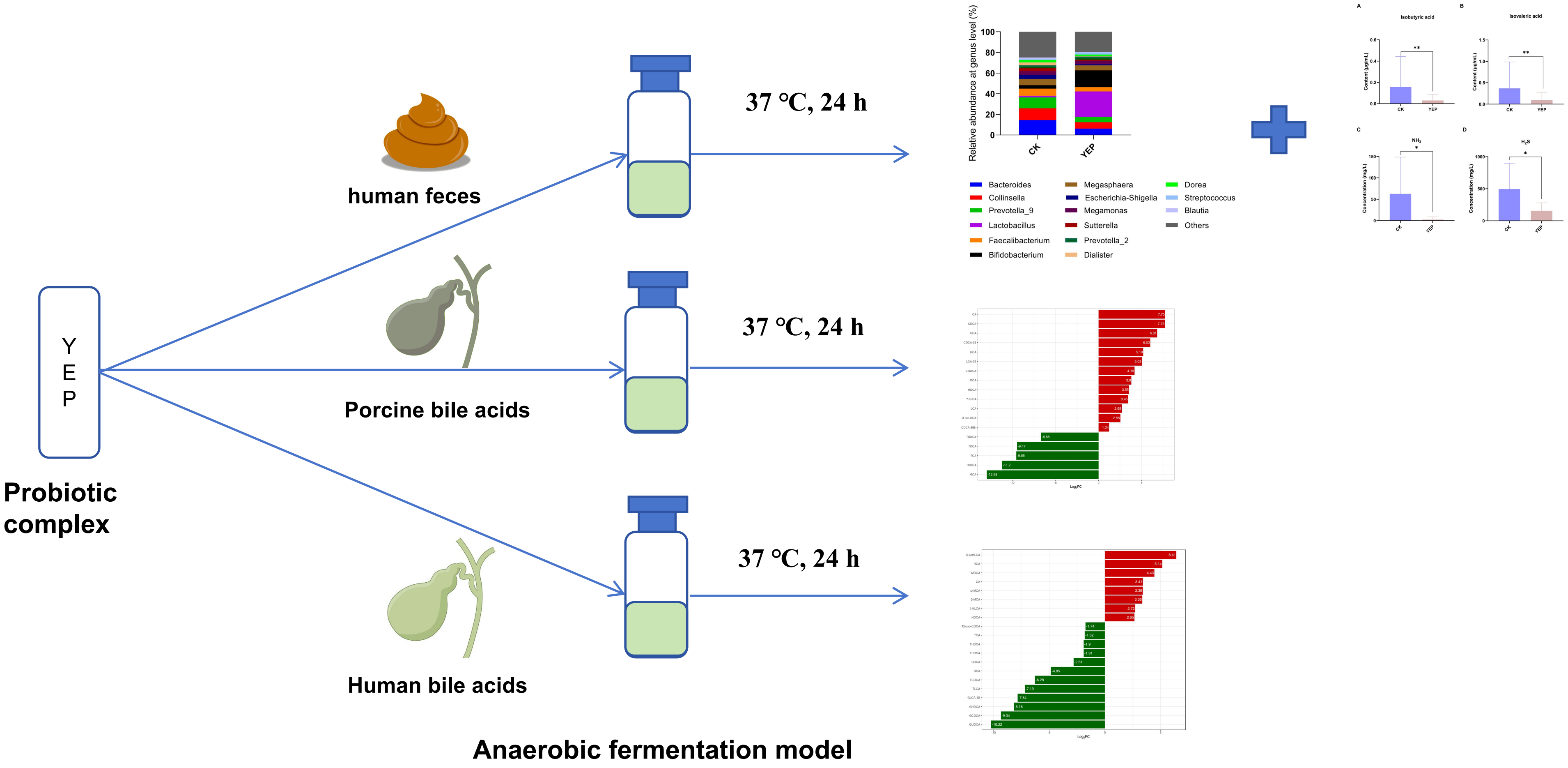

Supplement: Supplementary file 3 [file Data_Sheet_1.PDF]
